# Differential MicroRNA-Signatures in Thyroid Cancer Subtypes

**DOI:** 10.1155/2020/2052396

**Published:** 2020-06-03

**Authors:** Krystal Santiago, Yan Chen Wongworawat, Salma Khan

**Affiliations:** Center for Health Disparities & Molecular Medicine, Department of Pathology & Human Anatomy, Division of Biochemistry, Otolaryngology, Internal Medicine, Loma Linda University, School of Medicine, Loma Linda, CA, USA

## Abstract

Thyroid cancer is one of the most common endocrine cancers, with an increasing trend in the last few decades. Although papillary thyroid cancer is the most frequent subtype compared with follicular or anaplastic thyroid cancer, it can dedifferentiate to a more aggressive phenotype, and the recurrence rate is high. The cells of follicular adenomas and follicular carcinomas appear identical in cytology, making the preoperative diagnosis difficult. On the other hand, anaplastic thyroid cancer poses a significant clinical challenge due to its aggressive nature with no effective therapeutic options. In the past several years, the roles of genetic alterations of thyroid tumors have been documented, with a remarkable correlation between genotype and phenotype, indicating that distinct molecular changes are associated with a multistep tumorigenic process. Besides mRNA expression profiles, small noncoding microRNA (miRNA) expression also showed critical functions for cell differentiation, proliferation, angiogenesis, and resistance to apoptosis and finally activating invasion and metastasis in cancer. Several high-throughput sequencing studies demonstrate that miRNA expression signatures contribute clinically relevant information including types of thyroid cancer, tumor grade, and prognosis. This review summarizes recent findings on miRNA signatures in thyroid cancer subtypes.

## 1. Introduction

Thyroid cancer is the most common type of endocrine malignancy, and incidence has increased in the last few decades [1–4]. Although papillary thyroid cancer (PTC) is the most common differentiated thyroid cancer type, it is also known to have a good prognosis [5, 6]. PTC is the most common histologic type of differentiated thyroid cancer and comprises 75–85% of all thyroid malignancies in the United States [3]. Although PTC is curable, it can dedifferentiate into a more aggressive and lethal thyroid cancer characterized by metastasis to the cervical lymph nodes (LNs) [6]. According to the American Thyroid Association (ATA), thyroid sonography of cervical LNs should be performed in patients with known or suspected thyroid nodules before patients undergo a thyroidectomy [7–9]. The structure of LN metastasis differs from that of normal LN, which include cystic change, loss of normal architecture, calcification, and hyperechogenicity [10]. Because of its metastatic potential, intensive research has been done, and the diagnosis of thyroid cancer, particularly PTC, has increased immensely during the past three decades. Additionally, PTC recurs in approximately 30% of cases; therefore, it requires further molecular characterization.

Besides PTC, follicular thyroid cancer (FTC) is the other differentiated thyroid cancer [11, 12]. The cells from follicular adenomas (FA) and follicular carcinomas appear identical by cytology assay; cancer is diagnosed only by identifying capsular or vascular invasion on histologic specimens [13]. According to the *WHO Classification of Tumors of Endocrine Organs*, FTC is defined as a malignant epithelial tumor showing follicular cell differentiation and lacking the diagnostic nuclear features of PTC. Because of the difficulty of definitively diagnosing FTC, there has been an overdiagnosis of the follicular variant of papillary thyroid cancer [11]. Since FTC cannot be detected by cytology, diagnostic thyroid surgery is required, which shows a 20% positive malignant lesion out of all these surgeries [4]. According to the pattern of invasion, there have been two forms of follicular thyroid cancer identified, the minimally invasive and the widely invasive carcinomas. Fewer data are available on FTC and FA, so the goal of this review is to provide information on reliably discriminating FA from FTC. Mutation analysis showed that more than 30% of all FTC do not show any known mutations [14, 15]; therefore, the discovery of additional molecular markers is useful for the improvement of presurgical diagnosis of FTCs.

Anaplastic thyroid cancer (ATC) is another type of thyroid cancer with extremely poor prognosis [16–18]. ATC is an aggressive undifferentiated tumor, and because of its aggressiveness, the American Joint Committee classifies this cancer as a stage IV regardless of its tumor size, nodal status, or the absence or presence of distant metastases [18]. Although ATC is the rarest type of thyroid cancer, these characteristics make it contribute to 50% of the mortality from thyroid cancer. ATC poses a significant clinical challenge as it is highly aggressive, and there are no effective therapeutic options. Messenger RNA (mRNA) expression profiling demonstrates that ATC has a distinctive molecular signature [19]. Therefore, this systematic review adds information about the candidate biomarkers that are frequently expressed in ATC and may be used as potential diagnostic/prognostic biomarkers and therapeutic targets.

Lastly, medullary thyroid cancer (MTC) occurs in two forms: hereditary (HMTC) and sporadic (SMTC). Out of all, 75% of MTCs are sporadic and typically present at a late stage with nodal metastasis present at the time of diagnosis and a relatively poor prognosis.

In the past several years, knowledge of the genetic alterations of thyroid tumors has greatly increased. There is a remarkable correlation between genotype and phenotype, indicating that distinct molecular changes are associated with stages in a multistep tumorigenic process. Alterations of genes or gene pathways are linked to specific histologic features. This genotype/phenotype correlation provides the rationale for the numerous studies on gene expression profiling. The analysis of mRNA expression features may represent a useful tool for the preoperative diagnosis of thyroid nodules [20].

The translation from basic science to the potential diagnostic application also extends to recent studies on microRNAs (miRNAs) [21, 22]. These are small noncoding RNA molecules with critical functions for cell differentiation that negatively regulate gene expression, including known oncogene and tumor suppressor genes. Different subtypes of thyroid cancer are associated with specific miRNA profiles [23–31]. Technological advances allow the analysis of formalin-fixed routinely processed samples [32] as well as fine-needle aspiration biopsy (FNA) specimens using high-throughput sequencing [33], and as such their use as diagnostic and prognostic markers appears very promising. In this report, we have outlined the miRNAs involved in different subtypes of thyroid cancer from the existing literature in a comprehensive manner so that we can distinguish the subtype-specific miRNA signatures and follow their roles in tumor aggressiveness in future studies.

## 2. miRNAs

When miRNAs were discovered over 30 years ago, it was believed that they were conventional protein-coding genes. Yet after experimentation, Hammond made the remarkable discovery that instead of coding for protein, they represented a new class of long regulatory noncoding RNA which was later called miRNA [31]. miRNAs are 19–24 nucleotides long and bind to the 3′ UTR in messenger RNA to inhibit functional gene production. Their importance is attributed to their participation in development, cell proliferation, differentiation, apoptosis, and their role in many diseases including cancer [34]. Data also show that miRNAs can be used as potential biomarkers for the diagnosis and prognosis of a variety of diseases such as cancer, neurological disorders, cardiovascular diseases, and type-II diabetes; they can not only be used to monitor treatments but also for patient stratification [35, 36].

Differential miRNA expression profiles have been reported in tumors and normal tissues; such a phenomenon suggests the key role of miRNA in tumorigenesis. A single miRNA can have multiple targets with varying binding strengths. In addition to the amount and availability of the binding site, the binding strength determines whether miRNAs inhibit gene production by inhibiting translation or stimulating degradation of mRNA. Weak binding strength tends to inhibit the gene expression by targeting translation. mRNA instability and degradation most likely result when the corresponding miRNA sequence is completely complimentary, leading to tight binding and activation of the RNA-mediated interference (RNAi) pathway [37].

## 3. miRNAs and Cancer

There have been many studies that have identified the links between changes in miRNA expression from normal to cancer cells and tumor growth. With these findings and various tumors, miRNAs are currently being tested as potential diagnostic or prognostic biomarkers [38–41]. The most striking evidence that links miRNAs with cancer is their large alteration in expression in malignant cells compared to benign cells. miRNA expression is dysregulated in human cancer through various mechanisms, such as amplification or deletion of miRNA genes, abnormal transcriptional control of miRNAs, epigenetic changes, and defects in the miRNA processing machinery. Because of this dysregulation, miRNAs are classified as oncogenes or tumor suppressor genes. It is important to note that miRNAs are not always classified as tumor suppressors or as oncogenes; their classification depends on the specific tissue or cell type context in which they are expressed [41]. These genes regulate different carcinogenic processes in various types of malignancies [42]. The dysregulated miRNAs have an influence on the hallmarks of cancer, including proliferative signaling, evading growth suppressors, resisting cell death, inducing angiogenesis, activating invasion, and metastasis. Recent studies in cancer have shown that miRNAs play a very important role in cell differentiation; they can regulate the transformation of cancer cells and the acquisition of the epithelial-mesenchymal transition phenotype which is greatly associated with drug resistance [40]. Cancers including chronic lymphocytic leukemia, colon cancer, glioblastoma, and astrocytoma are associated with a change in the level of miRNA expression [43–48].

## 4. miRNA Expression Profile in Thyroid Cancer Subtypes

Numerous studies are going on to use miRNAs as potential biomarkers for human thyroid cancer diagnosis, prognosis, and therapeutic targets; these need further validation using a large cohort of samples. miRNAs are highly specific for a given cell and disease, relatively stable due to its shorter length (24 nucleotides) and therefore can get detected and reliably measured in various biological materials, including archived formalin-fixed and paraffin embedded samples or serum. With the availability of high throughput next-generation sequencing, miRNAs can be detected more abundantly and differentially from control compared with other miRNA microarrays, northern blots, TaqMan microRNA Assays Human panel, and confirmed by qRT-PCR assays. The miRNA expression profile presents a significant variability between different kinds of thyroid cancers, even if they originate from the same type of thyroid cells [21, 49]. For example, C-cell-derived medullary thyroid carcinoma has a miRNA expression profile significantly different from those of thyroid tumors originating from follicular cells. Although papillary carcinomas, conventional follicular adenomas and carcinomas, and oncocytic follicular adenomas and carcinomas all are originating from follicular cells, they show different and distinct miRNA expression profiles [50]. Although the exact biological roles of miRNAs in thyroid carcinogenesis remain to be fully studied, the specific miRNA expression profile in thyroid tumors compared to normal thyroid tissue may be useful in the diagnosis and treatment of thyroid neoplasia. Moreover, the different miRNA expression patterns in different types of thyroid tumors could be useful for their classification, serving as diagnostic markers and therapeutic markers [50, 51]. Analysis of miRNA expression levels and detection of circulating miRNAs can be used for the early diagnosis of thyroid cancer and for monitoring treatment responses [51].

In this review, we focus on miRNA expression in thyroid cancer to evaluate histological type-specific miRNA signatures, functional interactions with mRNAs, and miRNAs with the best potential as prognostic or diagnostic markers. Our goal is to provide a resource to strategize what future studies need to be performed. Although there are other review papers about miRNA and thyroid cancer subtype, such as review articles by Marini et al. and Celano et al. [50, 51], our review is more comprehensive and easier to understand by using Venn diagram. Venn diagram helps to illustrate upregulated and downregulated different miRNAs profile in different thyroid cancer subtypes and highlight the miRNAs that are in common. Moreover, our review especially focuses on the let-7 family and their expression profile in different thyroid cancer subtypes, including anaplastic thyroid cancer, which is not focused on other review papers to our best knowledge.

## 5. miRNAs in Papillary Thyroid Cancer

As papillary thyroid carcinoma (PTC) is the most common thyroid cancer, most miRNA expression research has been done in PTC cell lines or patient samples [23, 24, 26, 28, 32, 52–64]. A recent study showed the expression of miRNA-146b to be 28.9-fold higher in PTC tumors compared with control tissues [65]. Many other studies have found miR-146b expression upregulated in PTC [23, 28, 32, 57, 58, 66–68]. Various studies have found multiple functions of miR-146 including its roles in migration, invasion, proliferation, cell cycle, and chemotherapy resistance in BRAF-mutated cells [68–70]. Specifically, Deng et al. [69] found the Zinc Ring Finger 3 (ZNRF3) gene to be a direct target of miR-146b-5p leading to the downregulation of the gene product, resulting in ell migration, invasion, and epithelial-to-mesenchymal transition (EMT). More importantly, clinically, a study identified miR-146 as a possible prognostic biomarker, as they demonstrated increased miR-146 levels as an independent risk factor for worse prognosis in PTC patients [68].

Ab Mutalib et al. found miR-146b expression is significantly higher in PTC patients with lymph node metastasis positive (PTC LNM-P) or PTC patients with lymph node-negative (PTC LNN) fold change 6.0 and 4.7, respectively, when compared with adjacent normal thyroid tissue [71]. Additionally, other studies also found the miR-146b expression is greater in PTC LNM-P than in PTC LNN [52, 53, 56, 69]. Another miRNA that differed significantly in expression in PTC LNM-P compared with PTC LNN was miR-204. This miRNA was downregulated significantly in PTC LNM-P patients [71]. Additionally, miR-204 expression is downregulated in PTC versus adjacent normal thyroid tissue [24]. The miR-204, classified as a tumor suppressor miRNA, has been found to be downregulated in other cancers such as renal carcinoma [44], cervical cancer [45], and breast cancer [46]. Furthermore, in other cancer types, downregulated miR-204-5p has potential for use as a prognostic biomarker [47, 48]. As a tumor suppressor miRNA, Liu et al. found miR-204 in PTC cell lines inhibits cell proliferation, induces apoptosis, and arrests the cell cycle; additionally, miR-204 inhibited tumorigenicity in vivo [72].

Acibucu et al. and Yip et al. also found upregulation of miRNA 221 and miRNA 222 in PTC compared with normal thyroid tissue and in PTC with an increased risk of recurrence [56, 57]. Therefore, the more miRNA 146b, 221, and 222 are expressed, the higher the risk of recurrence of PTC because patients with upregulated expression of these miRNAs also had PTC that manifests with capsule invasion, vascular invasion, or lymph node metastasis. Of these 3 miRNAs, miRNA 146b was found to have a statistically significant difference in PTC with capsule invasion, vascular invasion, and lymph node metastasis. Similar to miRNA 146b, miRNA 221 and miRNA 222 are also involved in cell proliferation in thyroid cancer [23], and most notably upregulation of these miRNAs corresponds to increased tumor aggression [49]. In addition to normal thyroid tissue, another study found that miRNA 221 and 222 are upregulated in PTC compared with follicular adenoma and hyperplastic nodules [26, 66]. In agreement with Dai et al. also found miRNA 221 to be an independent risk factor for PTC recurrence [73].

Cantara et al. demonstrated that miR-95 and miR-190 are accurate and sensitive diagnostic markers for PTC in Caucasians [74]. Their results showed that miR-95 has a sensitivity of 94.9% and specificity of 98.7% in distinguishing PTC from nodular goiters and normal thyroid tissue. The sensitivity increases to 100% when miR-95 is combined with miR-190, which also has high diagnostic power. However, as this study only evaluated the diagnostic power of these two miRNAs in PTC, their diagnostic value can decrease depending on the expression pattern of miR-95 in other thyroid cancer types.

Another study [75] showed that miR-654-3p expression decreased in PTC over a long period of progression in vivo, which inversely correlated with EMT. The study then went on to demonstrate in vitro the tumor suppressor functions of miR-654-3p such as an increase in apoptosis and a decrease in cell proliferation, which has also been reported in prostate cancer cell lines [76]. Upon profiling serum miRNA in search of a serum biomarker for PTC, a pilot study [64] found 3 miRNAs that had statistically significant fold changes (5-fold change or more) between benign thyroid masses and PTC. One of the miRNAs was downregulated, miR-199b-3p, and the others were upregulated, let7b-5p and miR-10a-5p. According to Mohamad et al.'s study miR-205 is upregulated for PTC and miR-205 is associated with angiogenesis, proliferation, and invasion [77]. miRNA signature in PTC reported in the literature is shown in Table 1.

## 6. miRNAs in Follicular Thyroid Cancer

Besides PTC, follicular thyroid cancer (FTC) is the other type of differentiated thyroid cancer. In contrast to papillary thyroid cancer, follicular adenomas and carcinomas share the same tumorigenic pathway. Many studies have shown that the mRNA/miRNA expression profiles of follicular neoplasms differ from those of papillary carcinoma [86]; however, molecular signatures that clearly distinguish between follicular adenoma and follicular carcinoma need further validation in large studies. In a search for biomarkers to distinguish follicular thyroid cancer and PTC, dysregulated miRNA expression is well documented in follicular neoplasms. Samsonov et al. [86] found miRNA-21 and miRNA-181a-5p, which are exosomal miRNA, can differentiate the two cancer types with a sensitivity of 100% and specificity of 77%. Expression of miR-197 and miR-346 is significantly higher in follicular carcinoma than in follicular adenoma [21, 25, 27, 29, 49, 87].

In a meta-analysis study, miR-637, miR-181c-3p, miR-206, and miR-7-5p were revealed to be de novo potential FTC biomarkers; especially miR-7-5p showed the potential to distinguish between benign and malignant thyroid tissue in several published datasets [27]. Stokowy et al. [29] found two miRNA classifiers consisting of miR-484 and miR-148b-3p and distinguishes mutation-negative FTC from follicular thyroid adenomas with a sensitivity and specificity of 89% and 87%, respectively. In another study, Stokowy et al. [88] revealed different two miRNA classifiers consisting miR-7-5p and miR-7-2-3p for distinguishing PTCs and FTCs from benign thyroid masses. These two miRNA classifiers yielded a sensitivity and specificity of 82% and 49%, respectively.

Besides the miRNAs reported above, one study found that miR-146b, miR-183, and miR-221 (which are observed to be deregulated in PTC) are upregulated and miR-199b was downregulated in FTC versus normal thyroid cells [25].

Follicular variant of papillary thyroid carcinoma is the most common variant of PTC and is diagnosed in about 15–30% of PTC, sharing features of PTC and FTC on clinical, morphological, and genetic levels. It is important to distinguish between FTC and PTC with the follicular variant in clinical practice. Although miRNA deregulation was extensively studied in PTCs and FTCs, limited information is available for PTC with the follicular variant. Despite the high similarity in miRNA expression between PTC with the follicular variant and classical PTC, Dettmer et al. demonstrated that miR-375 is highly upregulated in PTC with the follicular variant and to a lesser extent in classic PTC, but not in FTC, hyperplastic nodule, or normal thyroid [30]. Besides, miR-181a-2-3p and miR-99b-3p can predict relapse-free survival in patients with PTC with follicular variant, thus may potentially provide important diagnostic and predictive value. miRNA signature in FTC reported in the literature is shown in Table 2.

## 7. miRNAs in Anaplastic Thyroid Cancer

Anaplastic thyroid carcinoma (ATC) is a highly malignant tumor. ATC is not beneficial in the radioactive scan but present with high mitotic activity in histologic appearance. miRNA profile showed significant downregulation of miR-30d, miR-30a-5p, miR-125b, and miR-26a expression. Out of these, a possible pathogenic role is suspected for miR-125b and miR-26a [91]. As seen in studies with PTC and FTC, miR-222 is deregulated in ATC. While miR-222 is upregulated in ATC, miR-25 is found to be downregulated in ATC cell lines compared with benign thyroid cells [92].

miR-30a and miR-200, two tumor suppressor miRNAs, are downregulated in ATC compared with the differentiated thyroid tumors and normal thyroid tissue [93, 94]. The downregulation of these miRNAs correlates with advanced tumor differentiation and increased tumor aggressiveness and mortality [93]. EMT has been implicated in ATC, and miR-30a normally inhibits invasion, migration, EMT markers, and lysyl oxidase (LOX) expression. Specifically, LOX inhibits cellular division, invasion, and migration in vitro and in vivo, demonstrating the potential for the miR-30a-LOX-axis as a target for ATC treatment [93]. The miR-200 family was recently identified as a suppressor of EMT and studies indicate a correlation between the downregulation of this miRNA family and the invasive potential of ATCs [95, 96]. The miR-200 family controls expression of essential EMT/mesenchymal-epithelial transition (MET) modulators, namely, ZEB1/2, SNAI2, and TGF*β*2, thus it is suggested to interfere with metastasis by sustaining an epithelial character of tissues [95–97].

Similar to miR-30a, miR-125b which is also a tumor suppressor miRNA, is downregulated in ATC. Another study suggests that the downregulation of miR-12b leads to PIK3CD upregulation, which is known to be involved in cell survival, growth, division, and glucose homeostasis [94, 98].

Family genes of let-7 are located on different chromosomes and are abundantly expressed in the normal thyroid gland (let-7a, let-7b, let-7c, let-7d, let-7e, let-7f, and let-7g) [99]. Deregulation of let-7 is observed in several types of cancer, and its tumor suppressor effects are usually abolished by its downregulation. Let-7 was found to be downregulated in ATC [94]. Downregulation of several members of the let-7 family is observed in well-differentiated thyroid cancer (PTC and FTC) [23, 24, 89, 90, 99], but a marked decrease in the expression of let-7a, let-7c, let-7d, let-7f, let-7g, and let-7i is also observed in ATC [26, 91, 100].

Fassina et al. [101] found that a 4-miRNA signature consisting of miR-26a, miR-146b, miR-221, and miR-222, is able to distinguish primary lymphomas from ATC with a sensitivity greater than 80% and a specificity greater than 90%. Clusters of miR 17–92 were found in ATC cells [102]. The upregulation of miR-20a was shown in ATC [103]. Upregulation of miR-146b, miR-221, and miR-222 and downregulation of miR-200 and miR-30 were reported [104]. Experimentally, it was shown that antisense-miR-21 enhances differentiation/apoptosis and reduces cancer stemness in ATC [105]. Mechanistically, it was shown that miR-99a inhibited tumorigenesis through targeting mTOR in ATC; on the other hand, miR-20a upregulation targeted LIMK1 in ATC. Therefore, strategy to restore tumor suppressor miRNAs and suppress the oncogenic miRNA will be a very promising therapeutic approach to ameliorate the treatment of ATC. miRNA signature in ATC reported in the literature is highlighted in Table 3.

## 8. miRNAs in Medullary Thyroid Cancer

In a miRNA microarray expression profile of a primary cohort of 12 SMTC and 7 HMTC samples, a number of miRNAs were identified to be differentially expressed [106, 107]. It was shown that overexpression of miR-183 and 375 in all MTCs predicted lateral lymph node metastases, residual locoregional disease, and mortality [106]. Pennelli et al. showed that miR-224 upregulation represents a prognostic biomarker associated with a better outcome in MTC patients [108]. Another group showed that, by array analysis, miR-200b and miR-200c are significantly downregulated in MTC metastases, consistent with reports showing downregulation of class of this miRNA during the metastatic process [109]. Hudson et al. reported that miR-375 and miR-10a were overexpressed, whereas miR-455 was underexpressed in MTC [110].

Chu et al. studied miR-21 and concluded that overexpression of miR-21 and MALAT1 regulate MTC progression and miR-21 inhibition reduced cell proliferation [111]. As mentioned above, many researchers have made it their mission to find the markers of metastatic cells and aggressive tumor behavior. Santarpia et al., went further to uncover 10 markers, two of which have been mentioned before, miR10a, miR-200b/-200c, miR-7, and miR-29c which were downregulated, whereas miR-130a, miR-138, miR-193a-3p, miR-373, and miR-498 were upregulated in MTC (or MTC progression?). These markers were involved in MTC pathways [109]. miRNA signature in MTC reported in the literature is depicted in Table 4.

## 9. Potential Therapeutic Implication of Targeting miRNA in Thyroid Cancer

miRNAs play a critical role in the regulation of various diseases, including thyroid cancer. Because the dysregulation of miRNA can be seen in many diseases, the possibility of using these as therapeutic agents has increased. According to Christopher et al., different laboratories have conducted recent studies in humans and animals. Their study shows us that miRNAs could become established as a new kind of drug [113]. Characteristics such as size and their conserved and known sequences make miRNAs attractive in terms of drug development. The effect that upregulated miRNA has in our biology can be eliminated by utilizing inhibitors or anti-miR. These are considered synthetic molecules that are used to prevent the miRNA from binding to its target. On the contrary, when miRNA levels are downregulated, it can be solved by targeting its positive regulation. An example of how this can be achieved is by delivering synthetic miRNA that imitates mature miRNAs. One of the approaches used to correct this anomalous miRNA expression is by synthesizing antisense oligonucleotides with a complementary sequence. These have been used to suppress dysfunctional mRNAs for more than four decades. It is widely recognized that the activation of oncogenes can lead to the formation of cancer, and to prevent this, artificial antisense miRNAs can be synthesized. By synthesizing this antisense oligonucleotide, it could be directed to block the miRNA that is causing cancer (known as oncomirs). However, vast knowledge must be known to use this technology. This includes the identity of the miRNAs that are dysregulated, their mechanism of action, applicability by RNAi, delivery of miRNAs, and their active form in vivo [113].

Another method that can be used to approach the therapeutic targeting of miRNAs is by expression vectors or miRNA sponges. This vector-based plan of action consists of introducing an mRNA that contains many binding sites for the specific miRNA that is dysregulated. Hence why it is considered a sponge. By overexpressing these mRNA-specific sponges, the miRNA that is causing the oncogenic or tumor-suppressive role binds to the specific sites in the sponge, thus liberating the target mRNAs and allowing their expression. Although many studies of this approach have been performed in vitro, only transgenic animals have been used for studies in vivo. The last approach taken in the therapeutic targeting of miRNAs is the usage of small-molecule inhibitors. According to Li and Rana these approaches utilize reporter-based assay systems for compound library screening, and they have identified small molecules that could specifically inhibit miRNA expression [114]. Instead of inhibiting target recognition, this approach focuses on the transcriptional regulation of these targeted miRNAs. However, the study specifies that the downfall of this approach is its high EC_50_ values (effector concentration for half-maximum response) and the lack of information on direct targets.

According to Wojcicka et al., no study has reported the clinical use of miRNAs for thyroid cancer treatment, although its strong potential has been mentioned in many functional and preclinical studies [115]. Most of these studies focus on PTC as it is the most common thyroid cancer. However, few to no studies focus on the potential of targeting miRNA on FTC and PDTC. The article specifies that miRNAs not only affect the regulation of tumor growth but they also influence the results of other therapies for thyroid cancer. Often, patients are not obtaining the desired results from the radioiodine therapies due to the lowered expression and function of the Na^+^/I^−^ symporter (NIS). It was discovered that the overexpression of miR-339 and miR-146b reduced the expression NIS in thyroid tumors. In contrast, the inhibition of these miRNAs allowed thyroid cancer cells to increase the uptake of radioactive iodine.

Various biotech and pharmaceutical companies have already started to develop products that use miRNAs therapeutics. However, they are focusing on miRNA mimics and antagomiRs. We have already established that miRNA mimics are used to re-establish the concentration of the specific miRNA that is downregulated and that is causing the pathology. In change, antagomiRs are used to repress those miRNAs that are upregulated and that are causing the pathology. According to Bonneau et al., there are two challenges that these companies have faced that must be managed for the success of this novel project [116]. Those are the stability and the delivery of the treatment. Contrary to DNA, RNA molecules are very unstable because they contain a 2′-OH chemical group. The other challenge, the delivery of RNAs to the targeted organs is necessary as it maintains the specificity of the treatment. Passive strategies such as the usage of the liver, the spleen, and the lymph nodes are used to internalize accumulated particles. Another strategy that could be used to deliver the therapy is through the active strategy. Here, the RNA or the particle with the specific molecule that will bind to the cell of interest will be endocytosed. There are no studies that elucidate the most favorable approach for thyroid cancer.

## 10. Conclusions

**Figure 1:**
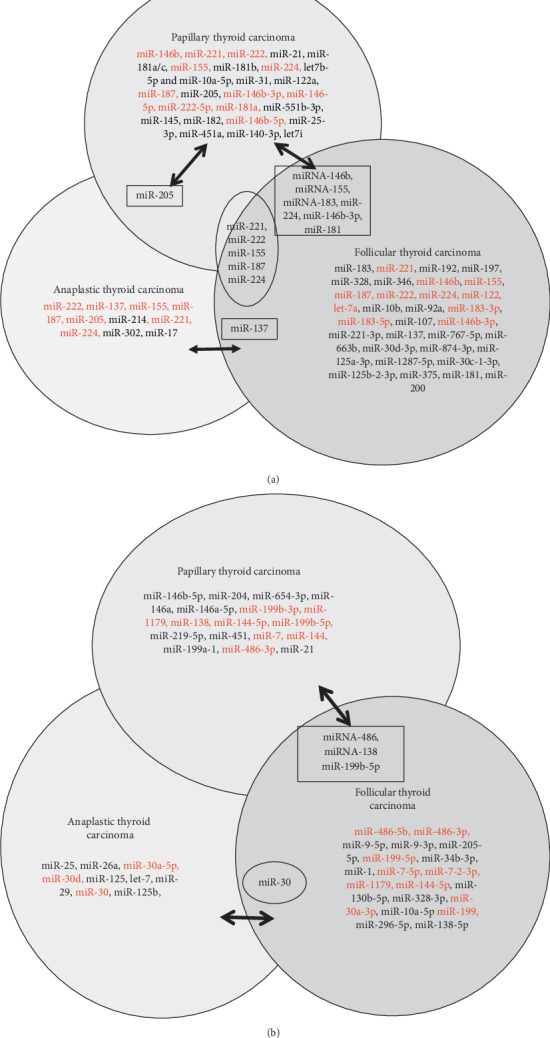
(a) Upregulated miRNAs. (b) Downregulated miRNAs.

In conclusion, we have summarized our literature search data in a Venn diagram to show the upregulated and downregulated gene pulls in subtypes of thyroid cancer (Figures 1(a) and 1(b)). We have also found very few common miRNAs that were upregulated/downregulated as shown in Figure 1. Most of the upregulated/downregulated miRNAs were subtype-specific, out of which miR-221, miR-222, miR-224, miR-155, and miR-187 were shown commonly in PTC, FTC, and ATC subtypes. Some of the miRNAs were common between PTC, and FTC, whereas very few overlapped between PTC to ATC (miR-205) and ATC to FTC (miR-137). However, we are not clear about the overlapping miRNAs or the subtype-specific miRNAs are important. Further studies need to clarify their roles.

With miR-146 being one of the most studied and most promising miRNAs involved with thyroid cancer, there is much interest in it as a gene target in hopes to reveal new prognostic biomarkers in PTC, especially those responsible for lymph node metastasis. Currently, more work is being done to profile miRNA in serum to find potential prognostic and diagnostic biomarkers. We suggest the need for more studies in vivo and multiple miRNA classifiers. Different studies have reported that high-throughput sequencing yielded more insight and better results in clarifying the relationship between miRNA and thyroid cancer [20, 24, 33]. The miRNA expression pattern is associated with tumor type, grade, and clinical outcomes. However, more efforts are still needed to screen miRNAs by deep sequencing in a large cohort of patient samples, especially for FTC and ATC. Though PTC is the most commonly diagnosed thyroid cancer, miRNA expression in ATC should be more thoroughly studied due to its large contribution to the mortality rate of thyroid cancers.

The limitation of our review article is that we did not perform meta-analysis; therefore, this lacks quantitative or qualitative analyses due to the limited number of literature on miRNA for each subtype of thyroid cancer and thus could not reveal the biases, strengths, and weaknesses of existing studies cited here. We also did not provide the mechanism of up and downregulation of miRNA-clusters and their pathways linked to each subtype-specific mRNA target. That would lead to another extended review on each subtype-specific pathway analysis, which awaits further ongoing extensive research.

Future studies are needed not only to identify novel miRNAs, but also their biological functions, roles in tumorigenesis, and ability to predict thyroid cancer prognosis, diagnosis, and treatment. In order to plan promising therapeutic strategies, it is necessary to investigate both upregulated and downregulated pulls of miRNA signatures and their mRNA targets in each of the thyroid cancer subtypes.

Recently, our lab focused on the miRNA signature in different ethnicities. However, in the literature search, we found that very few studies have been conducted to demonstrate the ethnic differences in miRNA expression in thyroid cancer health disparities [58, 74, 117]. Our lab has found a family of interesting miRNA signatures in Asian Americans with implications for thyroid cancer health disparities (unpublished data). Further studies are needed to demonstrate differential expression patterns and their mRNA targets to stratify the stress-induced mechanisms in cancer health disparities.

## Figures and Tables

**Table 1:** Up and downregulated miRNAs in papillary thyroid cancer.

Papillary thyroid cancer			
Upregulated miRNA	Ref	Downregulated miRNA	Ref
**miR-146b, miR-221, miR-222, miR-21** ^*∗*^ **, miR-181a/c** ^*∗*^ **, miR-155, miR-181b, miR-221, miR-224, let7b-5p, miR-10a-5p, miR-31, miR-122a, miR-146b, miR-155, miR-187, miR-205, miR-146b-3p, miR-146-5p, miR-222-5p, miR-181a, miR-551b-3p, miR-145, miR-182, miR-146b-5p, miR-25-3p, miR-451a, miR-140-3p, let7i**	[21, 23, 24, 28, 30, 49, 53–57, 62, 64, 67, 68, 78–84]	**miR-146b-5p** ^*∗∗*^ **, miR-204** ^*∗∗*^ **, miR-654-3p** ^*∗∗*^ **, miR-146a-5p** ^*∗∗*^ **, miR-199b-3p** ^*∗∗*^ **, miR-1179** ^*∗∗*^ **, miR-138, miR-144-5p** ^*∗∗*^ **, miR-199b-5p, miR-219-5p** ^*∗∗*^ **, miR-451** ^*∗∗*^ **, miR-7** ^*∗∗*^ **miR-144** ^*∗∗*^ **, miR-199a-1** ^*∗∗*^ **, miR-486-3p, miR-21** ^*∗∗*^	[24, 30, 52, 68, 72, 75, 78, 82, 83, 85]

^*∗*^miRNA targets genes by miRNA target screen: only specific upregulated miRNA and their target genes links in papillary thyroid cancer are underlined. http://www.mirdb.org/cgi-bin/search.cgi?searchType=miRNA&searchBox=hsa-miR-21-5p&full=1. http://www.mirdb.org/cgi-bin/search.cgi?searchType=miRNA&searchBox=hsa-miR-181a-5p&full=1. http://www.mirdb.org/cgi-bin/search.cgi?searchType=miRNA&searchBox=hsa-miR-181c-5p&full=1. http://www.mirdb.org/cgi-bin/search.cgi?searchType=miRNA&searchBox=hsa-miR-181b-5p&full=1. http://www.mirdb.org/cgi-bin/search.cgi?searchType=miRNA&searchBox=hsa-let-7b-5p&full=1. http://www.mirdb.org/cgi-bin/search.cgi?searchType=miRNA&searchBox=hsa-miR-10a-5p&full=1. http://www.mirdb.org/cgi-bin/search.cgi?searchType=miRNA&searchBox=hsa-miR-31-5p&full=1. http://www.mirdb.org/cgi-bin/search.cgi?searchType=miRNA&searchBox=hsa-miR-146a-5p&full=1. http://www.mirdb.org/cgi-bin/search.cgi?searchType=miRNA&searchBox=hsa-miR-222-5p&full=1. http://www.mirdb.org/cgi-bin/search.cgi?searchType=miRNA&searchBox=hsa-miR-551b-3p&full=1. http://www.mirdb.org/cgi-bin/search.cgi?searchType=miRNA&searchBox=hsa-miR-145-5p&full=1. http://www.mirdb.org/cgi-bin/search.cgi?searchType=miRNA&searchBox=hsa-miR-182-5p&full=1. http://www.mirdb.org/cgi-bin/search.cgi?searchType=miRNA&searchBox=hsa-miR-146b-5p&full=1. http://www.mirdb.org/cgi-bin/search.cgi?searchType=miRNA&searchBox=hsa-miR-25-3p&full=1. http://www.mirdb.org/cgi-bin/search.cgi?searchType=miRNA&searchBox=hsa-miR-451a&full=1. http://www.mirdb.org/cgi-bin/search.cgi?searchType=miRNA&searchBox=hsa-miR-140-3p&full=1. http://www.mirdb.org/cgi-bin/search.cgi?searchType=miRNA&searchBox=hsa-let-7i-5p&full=1. ^*∗∗*^miRNA targets genes by miRNA target screen: only specific downregulated miRNA and their target genes links in papillary thyroid cancer are underlined. http://www.mirdb.org/cgi-bin/search.cgi?searchType=miRNA&searchBox=hsa-miR-146b-5p&full=1. http://www.mirdb.org/cgi-bin/search.cgi?searchType=miRNA&searchBox=hsa-miR-204-5p&full=1. http://www.mirdb.org/cgi-bin/search.cgi?searchType=miRNA&searchBox=hsa-miR-654-3p&full=1. http://www.mirdb.org/cgi-bin/search.cgi?searchType=miRNA&searchBox=hsa-miR-146a-5p&full=1. http://www.mirdb.org/cgi-bin/search.cgi?searchType=miRNA&searchBox=hsa-miR-199b-3p&full=1. http://www.mirdb.org/cgi-bin/search.cgi?searchType=miRNA&searchBox=hsa-miR-1179&full=1. http://www.mirdb.org/cgi-bin/search.cgi?searchType=miRNA&searchBox=hsa-miR-144-5p&full=1. http://www.mirdb.org/cgi-bin/search.cgi?searchType=miRNA&searchBox=hsa-miR-219a-5p&full=1. http://www.mirdb.org/cgi-bin/search.cgi?searchType=miRNA&searchBox=hsa-miR-451a&full=1. http://www.mirdb.org/cgi-bin/search.cgi?searchType=miRNA&searchBox=hsa-miR-7-5p&full=1. http://www.mirdb.org/cgi-bin/search.cgi?searchType=miRNA&searchBox=hsa-miR-144-3p&full=1. http://www.mirdb.org/cgi-bin/search.cgi?searchType=miRNA&searchBox=hsa-miR-199a-5p&full=1. http://www.mirdb.org/cgi-bin/search.cgi?searchType=miRNA&searchBox=hsa-miR-21-5p&full=1.

**Table 2:** Up- and downregulated miRNAs in follicular thyroid cancer.

Follicular thyroid cancer			
Upregulated miRNA	Ref.	Downregulated miRNA	Ref.
**miR-146b, miR-183** ^*∗*^ **, miR-221, miR-192, miR-197, miR-328, miR-346, miR-146b, miR-155, miR-187, miR-222, miR-224, miR-122, let-7a, miR-10b, miR-92a, miR-183-3p, miR-183-5p, miR-107, miR-146b-3p, miR-221-3p, miR-137, miR-767-5p, miR-663b, miR-30d-3p, miR-874-3p, miR-125a-3p, miR-1287-5p, miR-30c-1-3p, miR-125b-2-3p, miR-375, miR-181, miR-200**	[25, 27, 29, 49, 78, 87–90]	**miR-486-5b, miR-486-3p, miR-9-5p** ^*∗∗*^ **, miR-9-3p** ^*∗∗*^ **, miR-205-5p** ^*∗∗*^ **, miR-199-5p, miR-34b-3p** ^*∗∗*^ **, miR-1** ^*∗∗*^ **, miR-7-5p, miR-7-2-3p, miR-11179, miR-144-5p, miR-130b-5p** ^*∗∗*^ **, miR-328-3p** ^*∗∗*^ **, miR-30a-3p, miR-10a-5p** ^*∗∗*^ **, miR-199, miR-296-5p** ^*∗∗*^ **, miR-138-5p** ^*∗∗*^	[25, 27, 78]

^*∗*^miRNA targets genes by miRNA target scan: only specific upregulated miRNAs and their target genes links in follicular thyroid cancer are underlined. http://www.targetscan.org/cgi-bin/targetscan.cgi?speciesHuman&mir_scmiR-183-5p.1. http://www.targetscan.org/cgi-bin/targetscan.cgi?speciesHuman&mir_scmiR-192-5p/215-5p. http://www.targetscan.org/cgi-bin/targetscan.cgi?species=Human&mir_nc=miR-197-3p. http://www.targetscan.org/cgi-bin/targetscan.cgi?speciesHuman&mir_cmiR-328-3p. http://www.targetscan.org/cgibin/targetscan.cgi?speciesHuman&gid&mir_sc&mir_c&mir_nc&mir_vnc&mirgmir-346. http://www.targetscan.org/cgi-bin/targetscan.cgi?speciesHuman&mir_vncmiR-10b-3p. http://www.targetscan.org/cgi-bin/targetscan.cgi?speciesHuman&mir_vncmiR-92a-1-5p. http://www.targetscan.org/cgibin/targetscan.cgi?speciesHuman&gid&mir_sc&mir_c&mir_nc&mir_vnc&mirgmir-107. http://www.targetscan.org/cgibin/targetscan.cgi?speciesHuman&gid&mir_sc&mir_c&mir_nc&mir_vnc&mirgmir-137. http://www.targetscan.org/cgibin/targetscan.cgi?speciesHuman&gid&mir_sc&mir_c&mir_nc&mir_vnc&mirgmir-767-5p. http://www.targetscan.org/cgibin/targetscan.cgi?speciesHuman&gid&mir_sc&mir_c&mir_nc&mir_vnc&mirgmir-663b. http://www.targetscan.org/cgibin/targetscan.cgi?speciesHuman&gid&mir_sc&mir_c&mir_nc&mir_vnc&mirgmir-30d-3p. http://www.targetscan.org/cgibin/targetscan.cgi?speciesHuman&gid&mir_sc&mir_c&mir_nc&mir_vnc&mirgmiR-874-3p. http://www.targetscan.org/cgibin/targetscan.cgi?speciesHuman&gid&mir_sc&mir_c&mir_nc&mir_vnc&mirgmiR-125a-3p. http://www.targetscan.org/cgibin/targetscan.cgi?species=Human&gid&mir_sc&mir_c&mir_nc&mir_vnc&mirgmiR-1287-5p. http://www.targetscan.org/cgibin/targetscan.cgi?speciesHuman&gid&mir_sc&mir_c&mir_nc&mir_vnc&mirgmiR-30c-1-3p. http://www.targetscan.org/cgibin/targetscan.cgi?speciesHuman&gid&mir_sc&mir_c&mir_nc&mir_vnc&mirgmiR-125b-2-3p. http://www.targetscan.org/cgibin/targetscan.cgi?speciesHuman&gid&mir_sc&mir_c&mir_nc&mir_vnc&mirgmiR-375. http://www.targetscan.org/cgi-bin/targetscan.cgi?species=Human&mir_sc=miR-181-5p. http://www.targetscan.org/cgi-bin/targetscan.cgi?species=Human&mir_sc=miR-141-3p/200a-3p. ^*∗∗*^miRNA targets genes by miRNA target scan: specific downregulated miRNAs and their target genes links in follicular thyroid cancer are underlined. http://www.targetscan.org/cgi-bin/targetscan.cgi?mirghsa-miR-486-5p. http://www.targetscan.org/cgi-bin/targetscan.cgi?mirghsa-miR-486-3p. http://www.targetscan.org/cgibin/targetscan.cgi?speciesHuman&gid&mir_sc&mir_c&mir_nc&mir_vnc&mirgmiR-199a-5p. http://www.targetscan.org/cgi-bin/targetscan.cgi?mirg=hsa-miR-7-5p. http://www.targetscan.org/cgi-bin/targetscan.cgi?mirg=hsa-miR-7-2-3p. http://www.targetscan.org/cgi-bin/targetscan.cgi?mirg=hsa-miR-144-5p. http://www.targetscan.org/cgi-bin/targetscan.cgi?mirg=hsa-miR-30a-3p. http://www.targetscan.org/cgi-bin/targetscan.cgi?mirg=hsa-miR-199a-3p.

**Table 3:** Up‐ and downregulated miRNAs in anaplastic thyroid cancer.

Anaplastic thyroid cancer			
Upregulated miRNA	Ref.	Downregulated miRNA	Ref.
**miR-222, miR-137, miR-155, miR-187, miR-205, miR-214** ^*∗*^ **, miR-221, miR-224, miR-302** ^*∗*^ **, miR-17** ^*∗*^	[49, 78, 91, 92]	**miR-25** ^*∗∗*^ **, miR-26a** ^*∗∗*^ **, miR-30a-5p, miR-30d, miR-125** ^*∗∗*^ **, let-7, miR-29** ^*∗∗*^ **, miR-30, miR-125b, miR-12b**	[78, 91–93, 98]

^*∗*^miRNA targets genes by miRNA target scan: only specific upregulated miRNAs and their target genes links in anaplastic thyroid cancer are underlined. http://www.targetscan.org/cgi-bin/targetscan.cgi?species=Human&mir_sc=miR-214-5p. http://www.targetscan.org/cgi-bin/targetscan.cgi?species=Human&mir_sc=miR-302-3p/372-3p/373-3p/520-3p. http://www.targetscan.org/cgi-bin/targetscan.cgi?species=Human&mir_sc=miR-17-5p/20-5p/93-5p/106-5p/519-3p. ^*∗∗*^miRNA targets genes by miRNA target scan: specific downregulated miRNAs and their target genes links in anaplastic thyroid cancer are underlined. http://www.targetscan.org/cgi-bin/targetscan.cgi?species=Human&mir_sc=miR-25-3p/32-5p/92-3p/363-3p/367-3p. http://www.targetscan.org/cgi-bin/targetscan.cgi?species=Human&mir_sc=miR-26-5p. http://www.targetscan.org/cgi-bin/targetscan.cgi?species=Human&mir_sc=miR-125-5p. http://www.targetscan.org/cgi-bin/targetscan.cgi?species=Human&mir_vnc=miR-125b-1-3p. http://www.targetscan.org/cgi-bin/targetscan.cgi?species=Human&mir_sc=miR-29-3p.

**Table 4:** Up- and downregulated miRNAs in medullary thyroid cancer.

Medullary thyroid cancer			
Upregulated miRNA	Ref.	Downregulated miRNA	Ref.
**miR-224, miR-375, miR-10a, miR-130a, miR-138, miR-193a-3p, miR-373, miR-1**	[107–110, 112]	**miR-200b** ^*∗∗*^ **, miR-200c** ^*∗∗*^ **, miR-21** ^*∗∗*^ **, miR-7** ^*∗∗*^ **, miR-29c** ^*∗∗*^	[109, 111]

^*∗*^miRNA targets genes by miRNA target scan: only specific upregulated miRNAs and their target genes links in medullary thyroid cancer are underlined. http://www.targetscan.org/cgi-bin/targetscan/vert_71/targetscan.cgi?mirg=hsa-miR-224-5p. http://www.targetscan.org/cgi-bin/targetscan/vert_71/targetscan.cgi?mirg=hsa-miR-375. http://www.targetscan.org/cgi-bin/targetscan/vert_71/targetscan.cgi?mirg=hsa-miR-10a-5p. http://www.targetscan.org/cgi-bin/targetscan/vert_71/targetscan.cgi?mirg=hsa-miR-130a-5p. http://www.targetscan.org/cgi-bin/targetscan/vert_71/targetscan.cgi?mirg=hsa-miR-138-5p. http://www.targetscan.org/cgi-bin/targetscan/vert_71/targetscan.cgi?mirg=hsa-miR-193a-3p. http://www.targetscan.org/cgi-bin/targetscan/vert_71/targetscan.cgi?mirg=hsa-miR-373-5p. http://www.targetscan.org/cgi-bin/targetscan/vert_71/targetscan.cgi?mirg=hsa-miR-1-5p. ^*∗∗*^miRNA targets genes by miRNA target scan: specific downregulated miRNAs and their target genes links in anaplastic thyroid cancer are underlined. http://www.targetscan.org/cgi-bin/targetscan/vert_71/targetscan.cgi?mirg=hsa-miR-200b-5p. http://www.targetscan.org/cgi-bin/targetscan/vert_71/targetscan.cgi?mirg=hsa-miR-200c-5p. http://www.targetscan.org/cgi-bin/targetscan/vert_71/targetscan.cgi?mirg=hsa-miR-21-5p. http://www.targetscan.org/cgi-bin/targetscan/vert_71/targetscan.cgi?mirg=hsa-miR-7-2-3p. http://www.targetscan.org/cgi-bin/targetscan/vert_71/targetscan.cgi?mirg=hsa-miR-29c-5p.
